# Counterfactual scenarios reveal historical impact of cropland management on soil organic carbon stocks in the United States

**DOI:** 10.1038/s41598-023-41307-x

**Published:** 2023-09-04

**Authors:** Stephen M. Ogle, F. Jay Breidt, Stephen Del Grosso, Ram Gurung, Ernie Marx, Shannon Spencer, Stephen Williams, Dale Manning

**Affiliations:** 1 Department of Ecosystem Science and Sustainability, Colorado State University, Fort Collins, CO 80523 USA; 2 Natural Resource Ecology Laboratory, Colorado State University, Fort Collins, CO 80523 USA; 3 Department of Statistics, Colorado State University, Fort Collins, CO 80253 USA; 4 Department of Statistics and Data Science, NORC at the University of Chicago, 55 East Monroe Street, Chicago, IL 60603 USA; 5 USDA-Agricultural Research Service, SMSBRU, Fort Collins, CO 80256 USA; 6 Department of Agricultural and Resource Economics, Colorado State University, Fort Collins, CO 80253 USA

**Keywords:** Environmental sciences, Climate change, Carbon cycle

## Abstract

Natural climate solutions provide opportunities to reduce greenhouse gas emissions and the United States is among a growing number of countries promoting storage of carbon in agricultural soils as part of the climate solution. Historical patterns of soil organic carbon (SOC) stock changes provide context about mitigation potential. Therefore, our objective was to quantify the influence of climate-smart soil practices on SOC stock changes in the top 30 cm of mineral soils for croplands in the United States using the DayCent Ecosystem Model. We estimated that SOC stocks increased annually in US croplands from 1995 to 2015, with the largest increase in 1996 of 16.6 Mt C (95% confidence interval ranging from 6.1 to 28.2 Mt CO_2_ eq.) and the lowest increase in 2015 of 10.6 Mt C (95% confidence interval ranging from − 1.8 to 22.2 Mt C). Most climate-smart soil practices contributed to increases in SOC stocks except for winter cover crops, which had a negligible impact due to a relatively small area with cover crop adoption. Our study suggests that there is potential for enhancing C sinks in cropland soils of the United States although some of the potential has been realized due to past adoption of climate-smart soil practices.

## Introduction

Reducing the influence of anthropogenic activity on the climate system is at the forefront of global discussions since the Paris Agreement was adopted at the COP21 of UN Framework Convention on Climate Change. Although fossil fuel combustion has been identified as the key driver of anthropogenic greenhouse gas (GHG) emissions and will need to be reduced to limit warming below the 2 °C goal^1^, natural solutions are likely also needed to achieve this goal^2, 3^. Enhancing soil C sinks in agricultural croplands is a natural solution proposed to contribute to this goal^4^, and sequestering C in soils is part of a larger plan for reducing GHG emissions by the national government in the United States^5^.

Soils contain a large pool of organic carbon and have been a significant source of CO_2_ emissions to the atmosphere since the advent of agrarian societies^6^. Climate-smart practices for soil management can enhance resiliency of agricultural systems and their sustainability, as well as mitigate GHG emissions and sequester C in soils. Practices that can enhance soil organic carbon (SOC) stocks include planting winter cover crops; reducing tillage intensity; rotating annual crops with perennials, such as years with hay or pasture; enhancing C input to soils through higher crop productivity with more productive varieties, irrigation and related practices; as well as adding amendments such as manure, compost, and biochar^7^. A recent global analysis suggests a technical potential for enhancing C sinks by 11.3 GtCO_2_ eq. year^−1^, and a cost-effective reduction of 5.3 GtCO_2_ eq. year^−1^ at a C price of $100 per t CO_2_ equivalent until 2050^8^. In this study, Roe et al.^8^ estimated an increase in SOC stocks for agricultural lands in the United States that would reduce atmospheric CO_2_ levels by 64 MtCO_2_ eq. year^−1^, excluding biochar amendments. This analysis focused on enhancement of C sinks with widespread adoption of winter cover crops and no-till management. In contrast, Fargione et al.^3^ estimated levels of SOC stock changes that would reduce atmospheric CO_2_ levels by 103 MtCO_2_ eq. year^−1^ based on winter cover crop adoption on 88 Mha of cropland in the United States.

Projections of GHG emissions mitigation scenarios are needed to quantify the potential to enhance soil C sinks, but the historical patterns of C sequestration in soils also provide context for assessing future potentials, i.e., a basis for evaluating the likelihood of achieving some level of mitigation given past patterns. Evidence of historical changes consistent with future projections suggest that the potential exists for agriculture to contribute meaningfully to mitigation goals. Past increases in SOC stocks from adoption of climate-smart soil practices, however, also imply that part of the potential to sequester C in soils has been realized. Moreover, historical context is needed given the uncertainty in achieving targets that have been suggested by programs such as the 4 per mille initiative^4, 9^.

Our objective was to implement a counterfactual analysis evaluating the historical influence of climate-smart soil practices on C sequestration in mineral soils that are used for crop production in the United States. To our knowledge, a counterfactual analysis has not been conducted for croplands in the United States to evaluate the historical impact of climate-smart soil practices on SOC stocks. Specifically, we conducted a model-based assessment using the DayCent Ecosystem Model, in which practices were removed from the historical time series in order to quantify their impact on the levels and trends in SOC stock changes. We focused on climate-smart soil practices that have been adopted in the past by farmers in the United States, including winter cover crop management, conservation tillage, hay and pasture in a rotation with annual crops, manure amendments, and setting-aside land from crop production.

## Results

### SOC stocks associated with climate-smart soil practices

**Figure 1:**
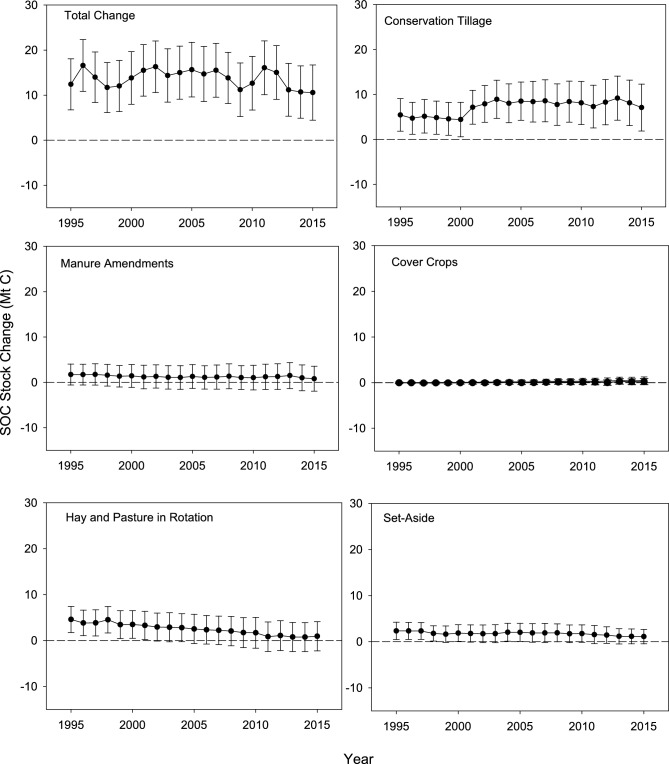
SOC stock changes (0–30 cm layer) and standard deviations (± 1 s.d.) from 1995 to 2015 and the contribution of management practices based on the counterfactual scenarios (Mt C). A positive value represents an increase in SOC stocks and a negative represents a decrease in SOC stocks.

Management of croplands on mineral soils in the United States has increased SOC stocks at varying levels over the time series, with the largest change of 16.6 ± 5.7 Mt C in 1996 and the lowest level of 10.6 ± 6.1 Mt C in 2015 (Fig. 1). The influence of practices varied over the time series with larger changes in SOC stocks from conservation tillage in the latter part of the time series, while manure amendments, hay and pasture in rotation with annual crops, and setting-aside land from production had a larger impact on SOC stocks in the early part of the time series. In contrast to these other practices, winter cover crops had a minimal effect on SOC stocks over the entire time series.

**Figure 2:**
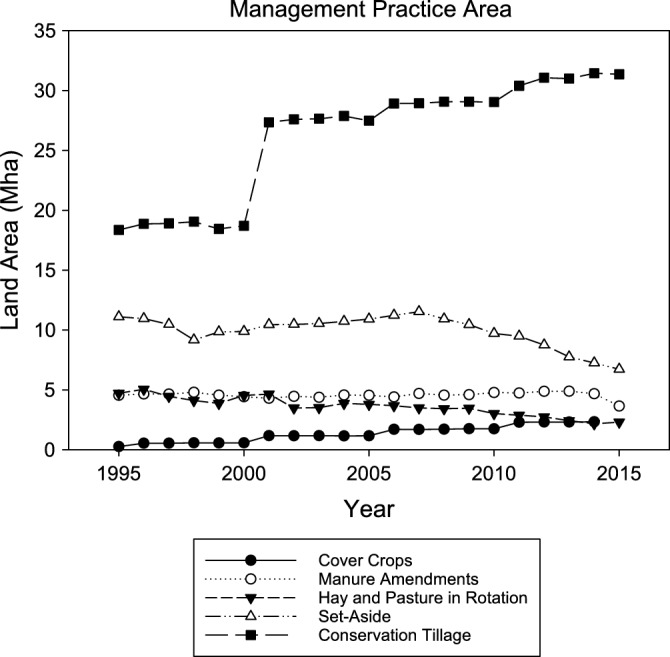
Trends in areas associated with winter cover crops, manure amendments, hay and pasture in rotation with annual crops, conservation tillage, and set-aside of cropland in reserve from 1995 to 2015 (Million ha).

**Figure 3:**
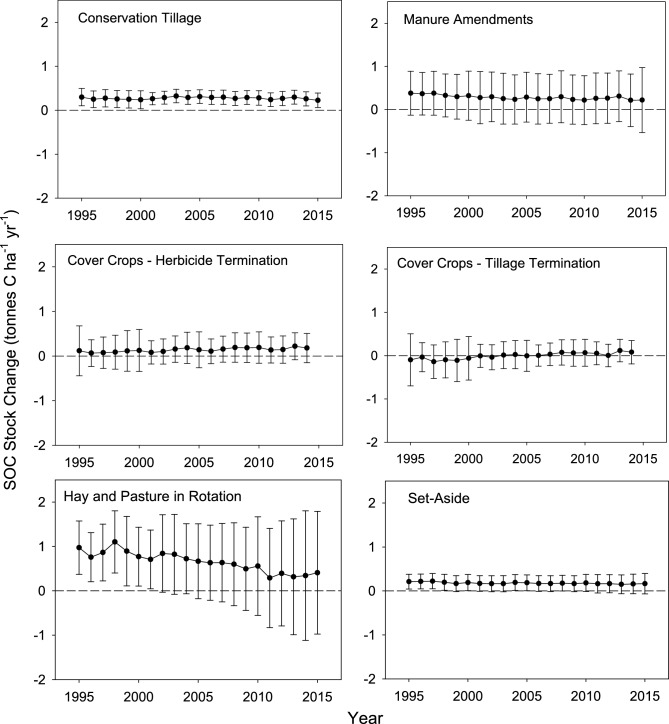
Trends and uncertainty (± 1 s.d.) in rates of SOC stock change (0–30 cm layer) associated with winter cover crops, manure amendments, hay and pasture in rotation with annual crops, conservation tillage, and set-aside of cropland in reserve from 1995 to 2015 (t C ha^−1^ year^−1^).

Conservation tillage was the only practice that increased the level of SOC stock change in the latter part of the time series. The underlying driver of this pattern was higher levels of adoption of conservation tillage starting in the early 2000s (Fig. 2) that led to the largest change in SOC stocks associated with the practice in 2013, estimated at 9.20 ± 4.9 Mt C according to the counterfactual analysis (Fig. 1). The rate of C sequestration on a per unit area basis across the time series varied between 0.23 ± 0.2 to 0.32 ± 0.2 t C ha^−1^ year^−1^ (Fig. 3).

In contrast to conservation tillage, the change in SOC stocks has remained relatively stable over time for manure amendments, and declined for hay and pasture in rotation with annual crops. The underlying drivers of these contrasting patterns were associated with trends in the areas of land managed with these practices and associated rates of C sequestration on a per unit area basis. Specifically, the area with manure amendments was relatively stable across the time series, averaging 4.5 Mha (Fig. 2), and the rate of C sequestration across the time series was also relatively stable, averaging 0.28 ± 0.6 t C ha^−1^ year^−1^ (Fig. 3). In turn, this led to an average change in SOC stocks of 1.3 ± 2.6 Mt C from 1995 to 2015 (Fig. 1). In contrast, the area with hay and pasture in rotation with annual crops declined from 4.7 Mha in 1995 to 2.3 Mha in 2015, and the rate of C sequestration decreased from 0.97 ± 0.6 t C ha^−1^ year^−1^ to 0.41 ± 01.4 t C ha^−1^ year^−1^. The decreasing rate of C sequestration occurred as the SOC pool approached a new equilibrium based on the level of C inputs and outputs in the model simulations^10^. Overall, the amount of SOC stock change associated with hay and pasture in rotation with annual crops was reduced from 4.6 ± 2.8 Mt C in 1995 to 0.94 ± 3.2 Mt C in 2015 (Fig. 1).

The US Department of Agriculture established a program to remove highly erodible land from production during the 1980s, known as the Conservation Reserve Program. Similar to hay and pasture in rotation with annual crops, the influence of setting-aside land from production also declined through time with the largest increase in SOC stocks of 2.4 ± 1.8 Mt C in 1996 to the lowest value of 1.1 ± 1.6 Mt C in 2015 (Fig. 1). The main driver of this pattern was the large decline in area enrolled in the Conservation Reserve Program over the time series (Fig. 2), while the rate of sequestration declined by a relatively small amount from 0.21 ± 0.2 t C ha^−1^ year^−1^ to 0.17 ± 0.2 t C ha^−1^ year^−1^ (Fig. 3).

**Figure 4:**
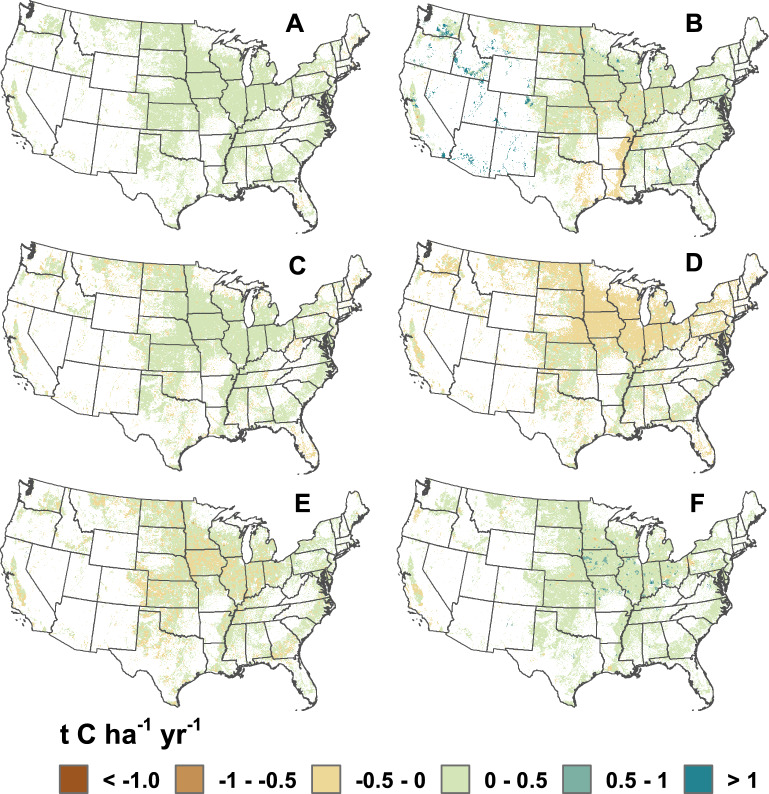
Average SOC stock changes (t C ha^−1^ year^−1^) from 1995 to 2015 on an interpolated 5 km grid for each of the management practices, including conservation tillage (**A**), manure amendments (**B**), winter cover crops with herbicide termination (**C**), winter cover crops with tillage termination (**D**), hay and pasture in rotation with annual crops (**E**), and setting-aside cropland from production (**F**). Maps produced in ArcGIS Release 10.7.1, https://www.esri.com.

Winter cover crops had a negligible influence on C sequestration due to a relatively small area managed with cover crops in the United States from 1995 to 2015 (Figs. 3 and 4). We did find that termination method for the cover crop, prior to planting the next crop, had a relatively large influence on the rate of C sequestration. There is currently insufficient data to determine termination practices, so we simulated two options, i.e., terminating the cover crop with tillage or with an herbicide application. Within the limited land base managed with winter cover crops, the rate of sequestration varied from a loss of SOC with tillage termination to gains with an herbicide termination (Fig. 3). Also, there is an underlying pattern of increasing stock change rates over 5-year time blocks because we simulated new adoption every 5 years. Future simulations could smooth this pattern with new adoption on an annual basis rather than 5-year time blocks.

### Spatial heterogeneity across the United States

There is spatial heterogeneity in the effect of management practices on SOC stock changes across the United States (Fig. 4). Averaging across the 21 years, incorporating hay and pasture in rotation with annual crops led to large variability in all regions ranging from change rates that were less than − 0.5 t C ha^−1^ year^−1^ to greater than 0.5 t C ha^−1^ year^−1^. In contrast, conservation tillage had relatively stable rates of SOC stock changes across the entire country, mostly in the range of no change to gains of 0.5 t C ha^−1^ year^−1^. As noted previously, the effect of cover crops is largely dependent on the termination method. With tillage termination, cover crop management led to losses of SOC in most of the north-central region of the United States, while the effect ranged from losses to increases in SOC for other regions. If cover crops were terminated with herbicides, then most of the croplands across the United States gained C, ranging from 0 to 0.5 t C ha^−1^ year^−1^. Similarly, manure amendments and setting-aside land from production typically ranged between no gain and 0.5 t C ha^−1^ year^−1^ averaged across the 21 years (Fig. 4). The exception was manure amendments in the south-central region where there is a relatively large area of losses, which appears to be associated with declining rates of manure amendments across the time series. There are also small areas in the western United States where manure amendments increased SOC stocks by larger rates exceeding 1 t C ha^−1^ year^−1^, and is related to relatively high amendment rates compared to other regions. Additional survey data on application practices are needed to confirm the rates of manure amendments in this region.

### Uncertainty

Since 1995, SOC stocks in mineral soils increased within the top 30 cm of the soil profile for cropland in the United States, with a high value in 1996 of 16.6 Mt C. The uncertainty in this estimate is relatively large with a 95% confidence interval ranging from 6.1 to 28.2 Mt C (Fig. 1) (Note: The uncertainty in Fig. 1 represents 1 s.d., but a 95% confidence interval is approximately 2 s.d.). The lowest estimate of SOC stock change is from 2015 with an increase of 10.6 Mt C, and again a relatively large 95% confidence interval ranging from − 1.8 to 22.2 Mt C. Furthermore, at a 95% confidence level, the uncertainties range over ± 100% for all practices across the time series, except for the impact of conservation tillage in 3 years (Fig. 1). While some of the uncertainty is associated with input data on management practices, most of the uncertainty is due to imperfect representation of processes in the DayCent model structure and associated parameterization, including soil organic matter dynamics, crop production, thermal regimes, and water flows through the crop-soil system (see Supplementary Information for more details about uncertainty in the DayCent model predictions).

## Discussion

The highest amount of SOC sequestration at 16.6 Mt C in 1996, which is 60.8 ± 21.1 Mt CO_2_ eq., is consistent with projections from Roe et al.^8^, who estimated a potential to reduce atmospheric CO_2_ levels by 64 MtCO_2_ eq. year^−1^ in the United States (Note: unit conversion into CO_2_ eq. to compare with estimates from Roe et al. study). However, the Roe et al.^8^ only focused on adoption of no-till management and winter cover crops. According to our analysis, there are approximately 30 Mha of cropland managed with conservation tillage so while there is potential for further adoption, this highlights that some of the potential has already been realized. In contrast, winter cover crops have not been adopted widely in the United States, so a limited amount of the potential has been realized. Limited adoption of practices such as winter cover crops may also imply that there is a higher cost associated with their adoption, and this could be a barrier to further adoption^11^.

Overall, the counterfactual analysis to the historical baseline of cropland management in the United States provides evidence that C can be sequestered in agricultural soils through climate-smart soil practices, such as conservation tillage, manure amendments, setting-aside land from cultivation, and incorporating hay and pasture in annual crop rotations. Cover crops may also sequester C in soils, but adoption rates will need to be considerably larger than historical levels to have much of an impact. Moreover, the effect will depend on the practice that is used to terminate the winter cover crop before planting the crop for the next growing season. There is less C sequestration if the cover crop is terminated with tillage rather than herbicides. Tillage is known to alter soil structure and the physical environment, impact microbial organisms, and enhance decomposition of soil organic matter^12–15^. This result is consistent with an empirical meta-analysis conducted by McClelland et al.^16^ who found less sequestration with tillage termination of cover crops.

Among these practices, adoption of conservation tillage is the most controversial^17, 18^. We found a modest level of sequestration at the national scale with average annual rates varying between 0.23 ± 0.2 to 0.32 ± 0.2 t C ha^−1^ year^−1^ across the time series to a 30 cm depth (Fig. 3). However, there are impacts on SOC stocks at deeper depths, particularly with no-till management^19–21^, leading to additional uncertainty in the effect of this practice. For example, Ogle et al.^22^ analyzed experimental data and found that no-till management could influence SOC stocks from 5 to 70 cm in temperate dry and wet climates depending on the soil characteristics, and Cai et al.^21^ found a significant impact to 60 cm overall without separating effects by climate and soil types. Future research and model development are needed to address effects deeper in the soil profile.

There have also been questions about the continuity of conservation tillage with potential for rotational tillage systems that incorporate a more intensive practice periodically. Recently, Lu et al.^23^ found a reduction in adoption of no-till management for corn-soybean rotations in the United States after 2008 due to herbicide resistance of weeds, and in turn, a loss of SOC after 2009 from cropland soils due to increasing tillage intensity. This contrasts with our findings, which showed a continuing trend of reducing tillage intensity in the latter part of the time series. We evaluated other crop systems besides corn and soybean rotations, and also included reduced tillage management in our study. It is noteworthy that Lu et al.^23^ used additional data sources from surveys conducted by a private firm called Kynetec that were not included in our analysis, and this may explain the difference between the two studies. We used data collected by the U.S. Department of Agriculture (USDA)^24, 25^ and the Conservation Technology Information Center^26^. Further analysis of the area of conservation tillage adoption is warranted in the future due to these inconsistencies. Regardless, given that some of the mitigation potential has been realized and probable challenges with continuous adoption of this practice, it is likely that conservation tillage may have a less significant role in reducing GHG emissions for the United States in the future.

Manure amendments can increase SOC stocks, and this practice had a relatively stable impact on SOC stocks over the time series. The potential could increase with additional livestock production, and more manure available for application to soils, but livestock production currently contributes significant amounts of GHG emissions, particularly CH_4_ emissions from enteric fermentation in ruminants^27^. Therefore, it seems unlikely that more livestock production to enhance soil C sinks would be strategic for meeting the goals of the Paris Agreement without the emergence of new tools and technologies that reduce GHG emissions from enteric fermentation and manure management. Independent of increasing livestock production, there is evidence that a more even distribution of crop and livestock systems across the country would increase the land base of manure amendments and may increase SOC stocks while reducing synthetic fertilizer inputs required to maintain yield levels^28^.

Hay and pasture in rotation with annual crops has increased SOC stocks historically, but the trend has been declining during the last decade. This trend is associated with less area in which hay and pasture are rotated with annual crops, in addition to declining rate of C sequestration on lands with this practice. Consequently, it may be that the role of incorporating hay and pasture into annual crop rotations will become less important to meet policy program goals, assuming there is limited new adoption of this practice over the historical baseline.

Setting-aside land from production can also increase SOC stocks (Fig. 1). Much of the land set-aside through the Conservation Reserve Program is marginal for crop production due to high erosion rates, and therefore less sustainable. These lands could be maintained in reserve or converted to grazing land, which would likely maintain the stocks over time. However, the area of reserve cropland has declined across the time series, leading to less removal of CO_2_ from the atmosphere. In addition, this analysis does not incorporate any leakage that may have occurred with setting aside land from agricultural production in the United States. For example, Wu^29^ estimated that for every 1 hectare of cropland in the United States that is placed in reserve, 0.2 hectare of non-cropland is converted to cropland. Land use conversion is likely to reduce SOC stocks^6, 30^, and counteract some of the benefit from setting-aside cropland into reserve.

Winter cover crop management is a candidate for further expansion in the United States, with current management on a relatively modest area of about 2 Mha (Fig. 2). Fargione et al.^3^ estimated reductions in atmospheric CO_2_ levels by 103 Mt CO_2_ eq. year^−1^ if this practice is adopted on 88 Mha. This would be approximately 75% of the United States cropland area included in our analysis. It is important to note that some regions in the United States have large amounts of winter grains, which would preclude adoption of winter cover crops, and may limit adoption to levels lower than proposed in the study by Fargione et al.^3^. It is noteworthy that the maximum simulated rates of C sequestration on a per unit area basis are about 0.22 t C ha^−1^ year^−1^ in our study, which is on the lower end of values estimated from meta-analyses of experimental data that range from about 0.1 to over 1 t C ha^−1^ year^−1^^16, 31^. Therefore, rates of C sequestration may be higher than we have simulated.

While meta-analyses are valuable for synthesizing results across studies, there are caveats with using them to infer dynamics across a country. In particular, meta-analyses are based on a convenience sample of data available in the literature^32^. These types of datasets may not provide an accurate statistical inference of the effect that cover crops, or other practices, have on SOC stock changes across the spatial domain of an entire country. A convenience sample may inadvertently under-represent or over-represent some cropping systems, soil types, other variables influencing soil organic matter dynamics, or even regions within the domain of a country, leading to biases in a resulting statistical inference. While weighting methods can help to reduce such biases, best practice would be based on a probability sampling design^33^ in which all cropland fields would have known, positive probabilities of selection in the sample. A national sample could be stratified to ensure representation across the range of factors affecting SOC stocks (soil and weather properties, management practices, etc.). Data from such a probability sample would be representative of all croplands and would support unbiased statistical inferences on SOC stock changes.

Regardless, given current rates of adoption, it is likely that expanding cover crop management could increase SOC stocks. There is evidence that adoption has increased over the past decade^34^, and is likely to continue increasing especially if there are effective programs to incentivize adoption. Assessing the effectiveness of such programs to enhance SOC is an active area of research. The USDA Environmental Quality Incentives Program (EQIP) and Conservation Stewardship Program (CSP) currently offer subsidies to encourage cover crop adoption. Park et al.^35^ found that the EQIP program increased the use of cover crops, but the CSP did not have the same impact according to their analysis. Programs designed to reduce GHG emissions must also account for the temporal variability in SOC changes, the potential lack of permanence that may occur with reversion of practice choices^36^, and the net effect of management changes on GHG emissions beyond the change in SOC stocks, such as the potential to increase soil N_2_O emissions^31, 37, 38^. In a recent example, Moore et al.^39^ accounted for impacts on soil N_2_O emissions as well as soil and biomass C storage, and estimated that adoption of conservation management practices in the United States has led to an average reduction of 134.2 Mt CO_2_ eq. year^−1^ based on data from 2017.

Uncertainty in quantifying the effect of climate-smart soil practices on an annual basis is also relatively large, even at the national scale, which will need to be factored into policy programs. At the scale of individual fields, uncertainties are further magnified ranging well over 100 percent^40^. The uncertainties are large because experimental site data, which are used to assess uncertainty (Supplementary Fig. S1), have a large range of variability, from gains to losses of SOC with adoption of climate-smart soil practices in some studies^41^. The DayCent ecosystem model does not always capture the range of these management effects. Further research and model development are needed to reduce these uncertainties, and there are promising avenues with new discoveries and paradigm shifts in our understanding of soil organic matter dynamics^42^. Moreover, there are limited observations for parameterizing models, highlighting the need to expand soil C monitoring networks through research alliances and other efforts to support assessments of SOC stock changes^43^.

The United States government is pledging to reduce GHG emissions by 50% over this decade in support of the Paris Agreement, and this includes C sequestration in agricultural soils as part of their larger plan^5^. Based on our counterfactual analysis, it is plausible that agricultural soils could sequester C and reduce atmospheric CO_2_ levels by the estimated 64 MtCO_2_ eq. year^−1^ from the Roe et al. study^8^. Climate-smart soil practices have been adopted historically in the United States and reduced atmospheric CO_2_ levels through sequestration of C in soils, but amounts have declined over time for several practices, including hay and pasture in rotation with annual crops, and setting-aside land from crop production. Further innovation may be possible in the agricultural sector to foster additional increases in SOC stocks, reduce other GHG emissions associated with management of agricultural lands, and make a more significant contribution to the Paris Agreement^44^. To the extent that history is an indicator of the future, agricultural management in the United States may be part of the climate solution, removing CO_2_ from the atmosphere by storing C in soils, but the historical level of C sequestration would only represent a small part of the total greenhouse gas emission reductions needed to achieve the goal of the Paris Agreement and limit warming below 2 °C.

## Methods

This assessment focuses on croplands with mineral soils (i.e., excludes *Histosols*) in the United States from 1995 to 2015 that were used to produce alfalfa hay, barley, corn, cotton, grass hay, grass-clover hay, oats, peanuts, potatoes, rice, sorghum, soybeans, sugar beets, sunflowers, tobacco, and wheat. These crops are grown on approximately 70% of total national cropland area. We excluded woody perennial crops and other less common annual crops on an area-basis, such as vegetables.

**Figure 5:**
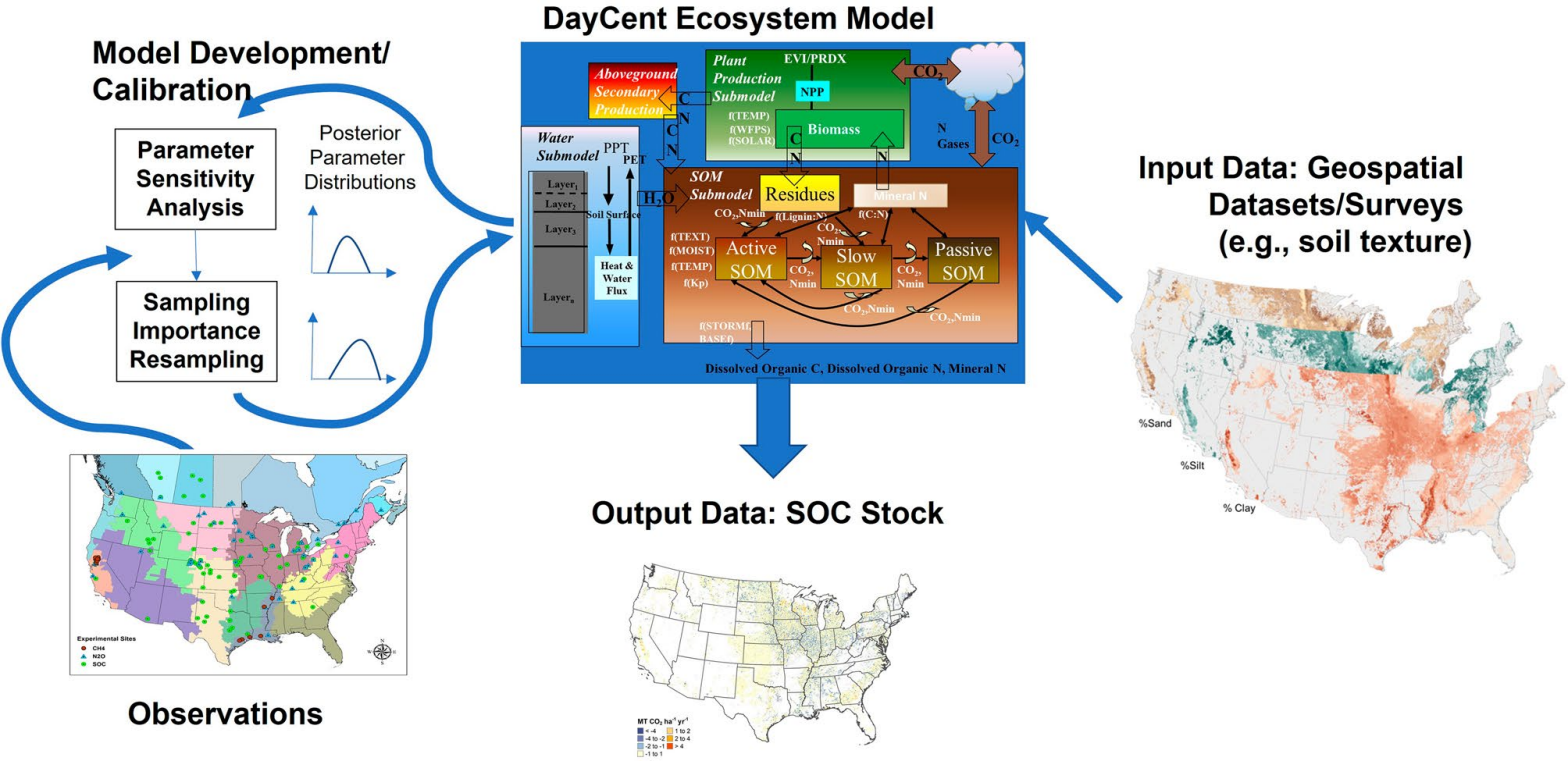
DayCent ecosystem modeling platform.

The assessment was conducted using a model-based platform with the DayCent Ecosystem Model (Fig. 5). We used the DayCent model because we have recently calibrated the model using experimental data^45^ and can quantify uncertainty to derive confidence intervals in the results^40^. DayCent is a process-based model simulating carbon and nitrogen dynamics in plant-soil systems on a daily time step to a 30 cm depth in the soil profile^45–49^. Input data included daily weather, edaphic characteristics, crop types and management information. The model has been calibrated with Bayesian methods using observations from long-term experiments. More information is given below about each of the platform components.

### Model calibration

The soil organic matter module of DayCent was calibrated using Bayesian methods^45^. First, a global sensitivity analysis was conducted with the Sobol method to select the most important model parameters influencing SOC dynamics in the model^50^. This method incorporates evaluation of interactions among the parameters to rank their influence on the predictions of SOC stocks. The global sensitivity analysis considered 17 model parameters and identified 9 parameters with total sensitivity indices > 2.5% for the final Bayesian calibration. The most sensitive parameters were drivers of SOC decomposition in the model simulations, including the decomposition rate constants, temperature effects, and tillage impacts on decomposition. Second, the most sensitive parameters were calibrated using the sampling importance resampling method^51^. This method approximates the joint posterior distribution of the parameters using a particle filter method based on the match between model output for each parameter set and observational data^52^. Model output that matches more closely to the measurements are given higher weights and are more likely to be selected in the resampling step. This method is non-iterative and explores the n-dimensional space of the parameters through a Monte Carlo analysis. More information about the sensitivity analysis and calibration are in Gurung et al.^45^. For application of DayCent in the model-based assessment of SOC stock changes, we used maximum a posteriori estimates of model parameters, which are the most likely parameter values from the joint posterior distribution. See Supplementary Information for evaluation of the SOC estimates from the DayCent model using independent sites from model calibration.

### Model input data

There are 4 general categories of model input data, including management practices, daily weather, edaphic characteristics and the enhanced vegetation index. The core dataset providing management information for this study was the USDA National Resources Inventory (NRI)^53, 54^. The NRI is a two-stage survey of land in the United States with primary sampling units that are stratified based on township and range from the United States Public Land Survey. The total cropland area in the assessment averaged 125.4 Mha from 1995 to 2015, with a high value of 128 Mha in 1995 and low of 124 Mha in 2012, and included an average of 171,397 survey locations from the NRI. Each survey location had a weight that is used to estimate the total amount and trend in SOC stock change for the land base. The NRI collects data using remote sensing technologies and site visits, and cropping histories were provided from 1979 to 2015.

Along with the cropping histories from the NRI, additional management data were needed to drive the model simulations, including synthetic fertilization, manure amendments, tillage practices, irrigation management, and winter cover crop management. Various data sources were combined to develop the time series of management practices (Table 1). Irrigation data were compiled through the NRI survey, but the remaining practices were imputed for the NRI locations using statistical methods. We created 6 imputation sets of management data in this process representing uncertainty in the assignment of practices to individual NRI survey locations.

**Table 1:** Data sources for management practices in United States agricultural croplands.

Management practice	Data source
Synthetic fertilization	1. USDA-NRCS Conservation Effects Assessment Project Survey (USDA-NRCS^24^ 2. USDA Agricultural Resource Management Surveys^55^ 3. Cropping Practices Survey^56^
Manure amendments	1. USDA-NRCS Conservation Effects Assessment Project Survey (USDA-NRCS^24^ 2. US-EPA Manure Management Database^57^
Tillage practices	1. USDA-NRCS Conservation Effects Assessment Project Survey (USDA-NRCS^24^ 2. USDA Agricultural Resource Management Surveys^25^ 3. Conservation Technology Information Center Data^26^
Irrigation management	1. USDA-NRI Survey^54^
Winter cover crops	1. USDA-NRCS Conservation Effects Assessment Project Survey (USDA-NRCS^24^ 2. USDA Census of Agriculture^58, 59^

Synthetic N fertilization and manure amendments were imputed for the NRI survey locations from the NRCS Conservation Effects and Assessment Project (CEAP) survey^24^. Synthetic fertilization practices were also informed by USDA-Economic Research Service datasets^55, 56^. For the imputation, trends in N management were determined based on the time series of fertilization rates from 1990 to 2015, with ERS Cropping Practices Survey data from 1990 to 1999, CEAP survey data from 2000 to 2005, and ARMS survey data for the remainder of the time series. The trends were determined at the scale of NRCS CEAP Regions^24^. With the regional trending information, an artificial neural network^60^ was used to identify likely N inputs at NRI survey locations within each CEAP region, and final N inputs for synthetic fertilization and manure amendments were assigned using a predictive mean matching method^61, 62^. The predictive mean matching method identified the most similar management activity recorded in the CEAP survey that matches the prediction from the artificial neural network. The matching ensures that imputed management activities were realistic for each NRI survey location, and not odd or physically unrealizable results that could be generated by the artificial neural network.

Historical trends in tillage management were based on data from the Conservation Technology Information Center (CTIC) for 1980 through 2004, CEAP survey for 2000 through 2005, and USDA Agricultural Resource Management (ARMS) surveys for 2002 through 2015^25^. The trends were determined at the scale of the CEAP regions^54^ in the 5-year time blocks from 1980 to 2015. In order to ensure time series consistency, a linear regression model was fit to model the CEAP tillage data as a function of the CTIC tillage data from 2001 to 2004, when there was overlap between the time series in these datasets. This regression model determined the relationship between CEAP and CTIC tillage estimates, and was used to adjust the CTIC tillage data in the 1980s and 1990s based on the relationship between the two datasets in the early 2000s. In addition, we used linear interpolation to gap-fill the missing 5-year time block from 2006 to 2010.

Tillage management practices, including continuous no-till (NT), reduced-till (RT) and full-till (FT), were imputed for each NRI survey location in 5-year blocks from 1980 to 2015. A tillage practice was assigned to each NRI locations in the imputation analysis using a hot-deck method (i.e., random selection from survey responses in the CEAP survey) for 2001–2005 with CEAP data aggregated by CEAP region, crop group, and soil texture class. Then, tillage management was imputed forward and backward from 2001 to 2005 using trending information in tillage management at the CEAP region scale (discussed above). This process used a novel, in homogeneous Markov–Chain approach to ensure a high degree of correlation in the type of tillage across time at each NRI survey location. For the first imputation step backward in time, the transition probability matrix for the Markov Chain was determined so that tillage type changed as little as possible (that is, NT-NT, RT-RT and FT-FT transitions were as likely as possible) subject to the constraint that the marginal proportions of NT, RT, FT at the start of the step (later time) matched the 2001–2005 time block, and the marginal proportions at the end (earlier time) matched the adjusted CTIC tillage data (linear regression model adjustment to ensure time series consistency between CTIC and CEAP data; see previous paragraph). Subsequent transition probability matrices further back in time then continued to enforce trending by matching the marginal proportions from the adjusted CTIC data. Similarly, transition probability matrices forward in time started with the CEAP data and then matched to the available ARMS data while ensuring as much stability as possible in the tillage types over time at each NRI survey location. The resulting imputed tillage types were sequences at each survey location that changed tillage type only rarely and at random, reflecting overall trends in tillage management practices.

Winter cover crop management data were compiled in the CEAP survey for 2000 through 2005 and USDA Census of Agriculture for 2012 and 2017^58, 59^. We used a hot deck method to impute winter cover crops for the NRI survey locations. First, we randomly assigned cover crops to NRI locations from 2001 to 2005 using survey responses in the CEAP data. Second, we assumed that winter cover crop management was negligible before 1990 and increased at a linear rate between 1990 to the 2000 levels from the CEAP survey. To represent this linear pattern, we randomly removed cover crops from NRI survey from 1996 to 2000 and then 1991 to 1995 reaching the target level of no cover crop management in 1990. For the time blocks after 2005 (i.e., 2006–2010 and 2011–2015), we randomly assigned or in some cases removed winter cover crops from NRI survey locations to match the trending information from USDA Census of Agriculture^58, 59^.

Additional model input data include daily weather, edaphic characteristics, and enhanced vegetation index data (EVI). Daily weather data are from a 4 km gridded weather data product produced by the PRISM Climate Group^63^. Edaphic characteristics, including soil texture, depth and pH, are assigned to each NRI survey location from the Soil Survey Geographic Database (SSURGO)^64^. The EVI data inform the estimation of net primary production in the DayCent model using the NASA-CASA production algorithm^65, 66^. The EVI time series is compiled from MODIS vegetation products (MOD13Q1 and MYD13Q1) with gap filling of the approximately 8-day intervals using the Savitzky–Golay Filter^67^. EVI data are available from 2000 to 2015 in our time series, and are used to inform production for corn, soybeans, sorghum, cotton, wheat, and other close-grown crops such as barley and oats. NPP is estimated using the standard crop production algorithm in DayCent for other crops and simulations prior to 2000^46^.

### Historical simulations and counterfactuals

Model simulations include an equilibrium and base history simulation to initialize the model’s state variables (e.g., initial levels of SOC in 1995), and then simulation of a historical assessment period from 1995 to 2015. The equilibrium simulation establishes steady-state conditions (e.g., equilibrium) under natural vegetation, historical climate data, and the soil characteristics for the NRI survey locations. We simulated the equilibrium conditions for 6000 years to achieve an approximate steady state in the DayCent model. For the first part of the base history, crop management was simulated with low input agriculture, i.e., no synthetic fertilization and pre-modern varieties, and the start of the base history simulations varied based on historical expansion of agriculture in the United States to 1950. In the second part of the base history from 1950 to 1979, we simulated increasing use of synthetic fertilizers and increasing productivity of crops that had occurred through breeding programs. The last part of the base history from 1980 to 1994 and the historical assessment from 1995 to 2015 were simulated with the data collected in the NRI survey and imputed information for management practices as discussed above.

The counterfactual scenarios were based on eliminating practices one at a time and quantifying the difference in SOC stock changes from the historical assessment simulation. The following counterfactual scenarios were simulated in this assessment:

- Winter cover crop management by eliminating cover crops in the model simulations;
- Conservation tillage by converting all reduced and no-till management systems to a full tillage system;
- Hay and pasture in a rotation with annual crops by replacing hay and pasture in the sequence with other annual crops in the time series for the NRI survey location (we replaced the hay and pasture by randomly selecting other annual crops grown at the survey location); and
- Manure amendments by changing the N input to synthetic mineral fertilization, and therefore not changing the amount of N available for crop production in the simulation.

The changes associated with the counterfactual scenarios were made in the early part of the NRI time series from 1979 to 1994, through the historical assessment period in 2015. We extended the management changes to 1979 in order to limit artificial trends in the C stocks during the assessment period that could have occurred if we abruptly shifted the management activity in 1995 for the historical time series in the counterfactual analysis.

The set-aside scenario was based on simulation of annual crops that are converted into grass cover when the land was set-aside from crop production according to the NRI survey data. No counterfactual case was simulated for set-aside management because the effect can be quantified directly from the historical simulation given that this is the only practice at the location (i.e., other practices, such as cover crop management, represent the combined effect of several practices in many cases, and so the impact of individual practices cannot be quantified directly from the historical simulation).

Spatial maps are provided for the average effect of climate-smart soil practices on SOC stocks from 1995 to 2015 on a 5 km grid (Fig. 4). The maps were created using an interpolation process with an inverse distance weighting spatial method that determines each grid cell value using a linear-weighted combination of sample points within a threshold distance of 20 km. NRI survey locations closer to the cell centroid were given more weight in calculating the SOC stock change than points further from the centroid. We required a minimum of 5 NRI survey locations within the 20 km threshold to calculate a grid cell value; otherwise, the grid cell was set to a null value.

### Error propagation and uncertainty

**Figure 6:**
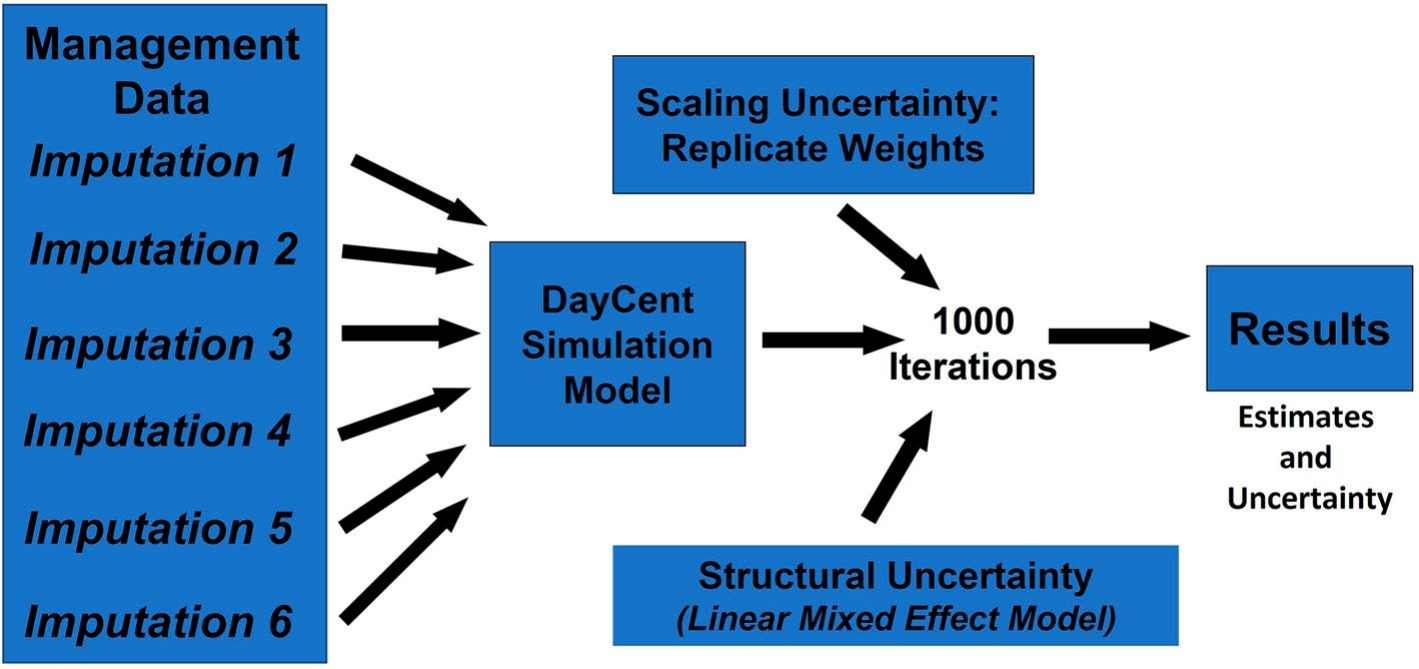
Monte Carlo analysis propagating uncertainty through the assessment associated with management data, model structure and parameterization based on an empirical function, and uncertainty in scaling results from NRI survey locations.

Uncertainty was quantified using a Monte Carlo approach adapted from Ogle et al.^40^ (Fig. 6). The sources of uncertainty include management input data, error in model structure and parameters, and scaling uncertainty associated with deriving total SOC stock change from the estimated changes at NRI survey locations. Uncertainty in the management data was determined by imputing 6 representations of the management histories for the NRI survey locations. Uncertainty in model structure and parameterization was approximated using an empirical approach with linear mixed effect model in which the ‘true’ SOC stocks, as measured in long term experiments, are modeled as a function of the predicted C stocks from DayCent and other covariates^68^. Random effects were included in these models to capture the dependence of data collected from the same experimental site and region. The resulting linear mixed effect model was applied to DayCent model output, adjusting for model bias and quantifying a level of precision in the model predictions (See Supplementary Information for more information). Scaling uncertainty was associated with estimating total SOC stock changes for the land base represented by the NRI sample in our analysis (averaging 171,397 survey locations across the time series). This uncertainty was quantified using replicate weights generated from the two-stage sampling design of the NRI survey and provided with the NRI dataset^54^. The replicate weights were used to approximate unbiased estimates of the variance that was associated with sampling error in the NRI (changes in the results if, hypothetically, repeated NRI samples were drawn from the population) and to approximate confidence intervals in the results.

The Monte Carlo analysis was conducted with 1000 iterations propagating uncertainty through the analysis from 3 major sources (Fig. 6). For each iteration, there was a random selection of DayCent model output based on one of the 6 imputations, a random selection of parameter values for the empirical estimator of structural and parameter uncertainty in the DayCent model; and a random selection of a set of replicate weights to scale SOC stock changes from the individual NRI survey locations to the entire domain of the assessment. Estimates were based on the mean and standard deviation from the simulated SOC stock changes across the Monte Carlo iterations.

### Supplementary Information

Supplementary Information 1.

Supplementary Information 2.

Supplementary Information 3.

## Data Availability

The observational data used to evaluate the DayCent Ecosystem Model are available in supplementary material. The spatial data from the counterfactual analysis are provided at the Dryad digital archive site (https://doi.org/10.5061/dryad.q2bvq83qx). The input data associated with the USDA-NRCS National Resources Inventory (NRI) and USDA-NRCS Conservation Effects Assessment Project are confidential and cannot be provided under United States Code, Title 18, Section  1905, United States Code, Title 7, Section 2276, and United States Code, Title 7, Section.  2204.
