# Supplementary material for: Counterfactual scenarios reveal historical impact of cropland management on soil organic carbon stocks in the United States
Source: Sci Rep. 2023 Sep 4;13:14564. doi: 10.1038/s41598-023-41307-x (PMC10477333; doi:10.1038/s41598-023-41307-x)
Supplement: Supplementary file 3 — Supplementary Information 3. [file 41598_2023_41307_MOESM3_ESM.docx]

**Supplementary Information**

**Counterfactual Scenarios Reveal Historical Impact of Cropland Management on Soil Organic Carbon Stocks in the United States**

Stephen M. Ogle, F. Jay Breidt, Stephen Del Grosso, Ram Gurung, Ernie Marx, Shannon Spencer, Stephen Williams, and Dale Manning

This supplement provides additional results from an evaluation of the SOC predictions by the DayCent Model based on the calibration published by Gurung et al. (2020). Data were compiled from 52 long-term cropland experiments with 393 observations of SOC, representing a variety of crops and management practices (Supplementary Table S1, SOCLongTermExperiments.xlsx). There were additional sites used to calibrate the DayCent model, which are presented in Gurung et al. (2020), including Broadbalk plots at Rothamsted Experimental Site, England; Cheyenne, Wyoming, USA; Grignon, France; Mead, Kansas, USA; Pendleton, Oregon, USA; South Charleston, Ohio, USA; Swift Current, Saskatchewan, Canada; Wooster, Ohio, USA. In addition to the experimental studies, the evaluation dataset included another 163 observations collected from 123 survey locations in the USDA-NRCS National Resources Inventory (NRI) (SOCSoilMonitoringNetwork.xlsx), (USDA-NRCS 2018a). Data were collected from the NRI sites using the methods described in Spencer et al. (2011).

Supplementary Table S1. List of experimental studies used to evaluate the DayCent Model estimates of SOC, along with years, management practices, measured and modeled SOC.

| **Location** | **Studies** | **Years of Experiment** | **Management Practice Effects** |
| --- | --- | --- | --- |
| Akola, India | Manna et al. 2007 ;  Bhattacharyya et al. 2010 | 1988-2001 | Organic and inorganic N fertilizer sources |
| Arlington, Wisconsin, USA | Vanotti et al. 1997 ;  Collins et al. 2000 | 1958-1993 | Inorganic N fertilizer levels |
| Arlington, Wisconsin, USA | Brye and Kucharik 2003 | 1976-2000 | Cropland vs. Restored grassland |
| Arvada, Wyoming, USA | Reeder et al. 1998 | 1987-1993 | Cropland vs. Set-aside land from production |
| Atwood, Kansas, USA | Gebhart et al.1994 | 1987-1992 | Cropland vs. Set-aside land from production |
| Bad Lauchstädt, Germany | Powlson et al. 1998 ;  Ludwig et al. 2007 | 1902-1992 | No fertilizer vs. farmyard manure |
| Beijing Province, China | Zhang et al. 2009 | Early 1990s-2005 | Low, medium and high input crop systems |
| Big Spring, Texas, USA | Gebhart et al. 1994 | 1987-1992 | Cropland vs. Set-aside land from production |
| Brazos River floodplain, Texas, USA | Franzluebbers et al. 1995 ;  Dou et al. 2007 | 1982-2002 | Rotations |
| Breton, Alberta, Canada | Juma et al. 1997; Izaurralde et al. 2001 | 1930-1992 | N fertilizer sources, and Rotations |
| Burleson, Texas, USA | Potter et al. 1999 | 1972-1998 | Cropland vs. Restored grassland |
| Bushland, Texas, USA | Paustian and Elliott, unpublished data | 1981-1993 | Tillage intensity, and Rotations |
| Calloway County, Missouri, USA | Udawatta et al. 2008 | 1991-2005 | Cropland, Set-aside land from production, Restored grassland |
| Colby, Kansas, USA | Gebhart et al.1994 | 1987-1992 | Cropland vs. Set-aside land from production |
| Davis, California, USA | Clark et al. 1998 | 1988-1996 | 2-year and 4-year rotation |
| Delhi, Ontario, Canada | Wanniarachchi et al. 1999 | 1988-1994 | Tillage intensity |
| Dixon Springs, Illinois, USA | Kitur et al. 1994 ;  Olson et al. 2010 | 1989-2009 | Tillage intensity |
| East Lansing, Michigan, USA | Pierce and Fortin 1997;  Paustian and Elliot, unpublished data | 1980-1992 | Tillage intensity and Cover crops |
| Eldorado do Sul, Brazil | Bayer et al. 2000 | 1983-1994 | Rotations |
| Fermilab, Batavia, Illinois, USA | Matamala et al. 2008 | 1975-1997 | Cropland vs. Restored prairie |
| Grant County, North Dakota, USA | Bauer and Black 1992 | 1916-1979 | Soil texture differences with same management |
| Harare, Zimbabwe | Chivenge et al. 2007 | 1988-1998 | Tillage intensity with Soil texture differences |
| Horseshoe Bend, Georgia, USA | Paustian and Elliott, unpublished data | 1978-1992 | Tillage intensity |
| Hoytville, Ohio, USA | Dick et al. 1997 ;  Collins et al. 2000 | 1963-2005 | Tillage intensity, and Rotations |
| Indian Head, Saskatchewan, Canada | Paustian and Elliot, unpublished data | 1957-1992 | Rotations with different levels of fallow frequency, and Inorganic N fertilizer levels |
| Kellogg Biological Station, Hickory Corners, Michigan, USA | Collins et al. 2000 | 1989-1993 | Tillage intensity, and Inorganic N fertilizer levels |
| Keeline, Wyoming, USA | Reeder et al. 1998 | 1987-1993 | Cropland vs. Set-aside land from production |
| Lamberton, Minnesota, USA | Huggins and Fuchs 1997; Collins et al. 2000 | 1960-1992 | Inorganic N fertilizer levels, timing and rate |
| Lancaster, Wisconsin, USA | Vanotti et al. 1997 | Circa 1861- 1966 | Pre-experiment sample |
| Lancaster, Wisconsin, USA | Karlen et al. 1994 | 1978-1991 | Tillage intensity and Residue management |
| Lethbridge, Alberta, Canada | Hao et al 2003 | 1973-1998 | Manure application rates and Irrigation |
| Lethbridge, Alberta, Canada | Monreal and Janzen 1993 | 1910-1990 | Fallow frequency |
| Lethbridge, Alberta, Canada | Janzen et al. 1997 | 1970-1992 | Tillage intensity |
| Lethbridge, Alberta, Canada | Janzen et al. 1997 | 1951-1992 | Fallow frequency, and Inorganic N fertilizer levels |
| Lexington, Kentucky, USA | Frye and Blevins 1997;  Elliott et al. 1994 | 1970-1992 | Tillage intensity, and Inorganic N fertilizer levels |
| Mandan, North Dakota, USA | Black and Tanaka 1997 | 1984-1991 | Tillage intensity, Inorganic N fertilizer levels, and Rotations |
| Manhattan, Kansas, USA | Havlin and Kissel 1997 | 1974-1992 | Tillage intensity, and Rotations |
| Melfort, Saskatchewan, Canada | Paustian and Elliott, unpublished data | 1957-1992 | Rotations, and Inorganic N fertilizer levels |
| Morrow plots, Champaign-Urbanna, Illinois, USA | Khan et al. 2007 | 1955-1991 | Rotation, and Inorganic N fertilizer levels |
| Otis, Colorado, USA | Denef et al. 2008 | 1979-2005 | Center pivot irrigation |
| Parkgrass plots, Rothamsted, England | Poulton 1996;  Rothamsted Research 2015 | 1856-2002 | Organic and Inorganic N fertilizer sources |
| Riesel, Texas, USA | Potter et al. 1999 | 1880-1998 | Cropland vs. Restored grassland |
| Rodale, Kutztown, Pennsylvania, USA | Doran et al. 1987;  Paustian and Elliott, unpublished data | 1981-1992 | Conventional grain systems with inorganic N fertilizer and herbicides; Grain crops with legumes in rotation with no inorganic N fertilizer; Grain and forage rotation with legumes and manure amendments and no inorganic N fertilizer |
| Rosemount, Minnesota, USA | Clapp et al. 2000 ;  Dolan et al. 2006 | 1980-2002 | Residue management, Tillage intensity, and Inorganic N fertilizer levels |
| Saginaw Valley, Michigan, USA | Christenson 1997;  Collins et al. 1999 | 1972-1992 | Rotations |
| Seminole, Texas, USA | Gebhart et al.1994 | 1963-1992 | Cropland vs. Set-aside land from production |
| Sidney, Nebraska, USA | Follett et al. 1997 ; Elliott et al. 1994 | 1970-1993 | Tillage intensity |
| Sterling, Colorado, USA | Peterson and Westfall 1997;  Paustian and Elliott, unpublished data | 1985-1992 | Rotations on 3 Landscape positions |
| Stratton, Colorado, USA | Peterson and Westfall 1997; Paustian and Elliott, unpublished data | 1985-1992 | Rotations on 3 Landscape positions |
| Syracuse, Nebraska, USA | Reedy 1999; Martens et al. 2003 | Circa 1866-1996 | Cropland vs. Pasture |
| Temple, Texas, USA | Potter et al. 1999 | 1992-1998 | Cropland vs. Restored grassland |
| Treynor, Iowa, USA | Manies et al. 2001 | Circa 1860-1998 | Cropland vs. Grassland |
| Valentine, Nebraska, USA | Gebhart et al.1994 | 1979-1992 | Cropland vs. Set-aside land from production |
| Versailles, France | Bol et al. 2008 | 1929-2008 | Organic and inorganic N fertilizer sources to bare fallow |
| West Lafayette, Indiana, USA | Elliott et al. 1994; Paustian and Elliott, unpublished data | 1975-1992 | Tillage intensity |
| Woodslee, Ontario, Canada | Yang et al. 2008 | 1982-1998 | Tillage intensity |

^a^ SOC values to a 30 cm depth were approximated based on an exponential decrease in the C stock values in shallower depths. Only studies with a measurement to 20 cm were included in the analysis, and at least 2 depth increments were required to approximate the SOC to a 30 cm depth.

The estimated SOC values from DayCent were evaluated by fitting a linear mixed effect model in which the observed SOC stocks were modeled as a function of the modeled SOC stocks and a covariate for grassland versus cropland land use. Data were transformed with a natural log to meet model assumptions of equality of variance and a normal distribution of the error. Random effects were assigned to each experimental site/NRI survey location, and to data from the same region following the classification for the USDA-NRCS Conservation Effects Assessment Project (CEAP) (USDA-NRCS 2019b). Sites outside of the United States were identified as ‘other’ for the region designation. The two hierarchical levels in the random effects capture correlation in observations from the same site and region. Figure S1 presents the modeled verses observed SOC stocks and the fitted linear mixed effect model. The linear-mixed effect model is used to adjust for model bias and quantify the level of precision in DayCent estimates for the national inventory, incorporating uncertainty in the fixed effects, random effects, and residual error.

**Supplementary Figure S1.** Measured SOC from experiment study sites and NRI survey sites as a function of the DayCent model estimates. The fitted line is the linear mixed-effect model in which the natural log of observed SOC stocks (ln(obs)) are modeled as a function of natural log of the modeled SOC stocks (ln(mod), random effect for experimental/NRI site (rsite), random effects for region (rceap), and remaining residual error (resi).


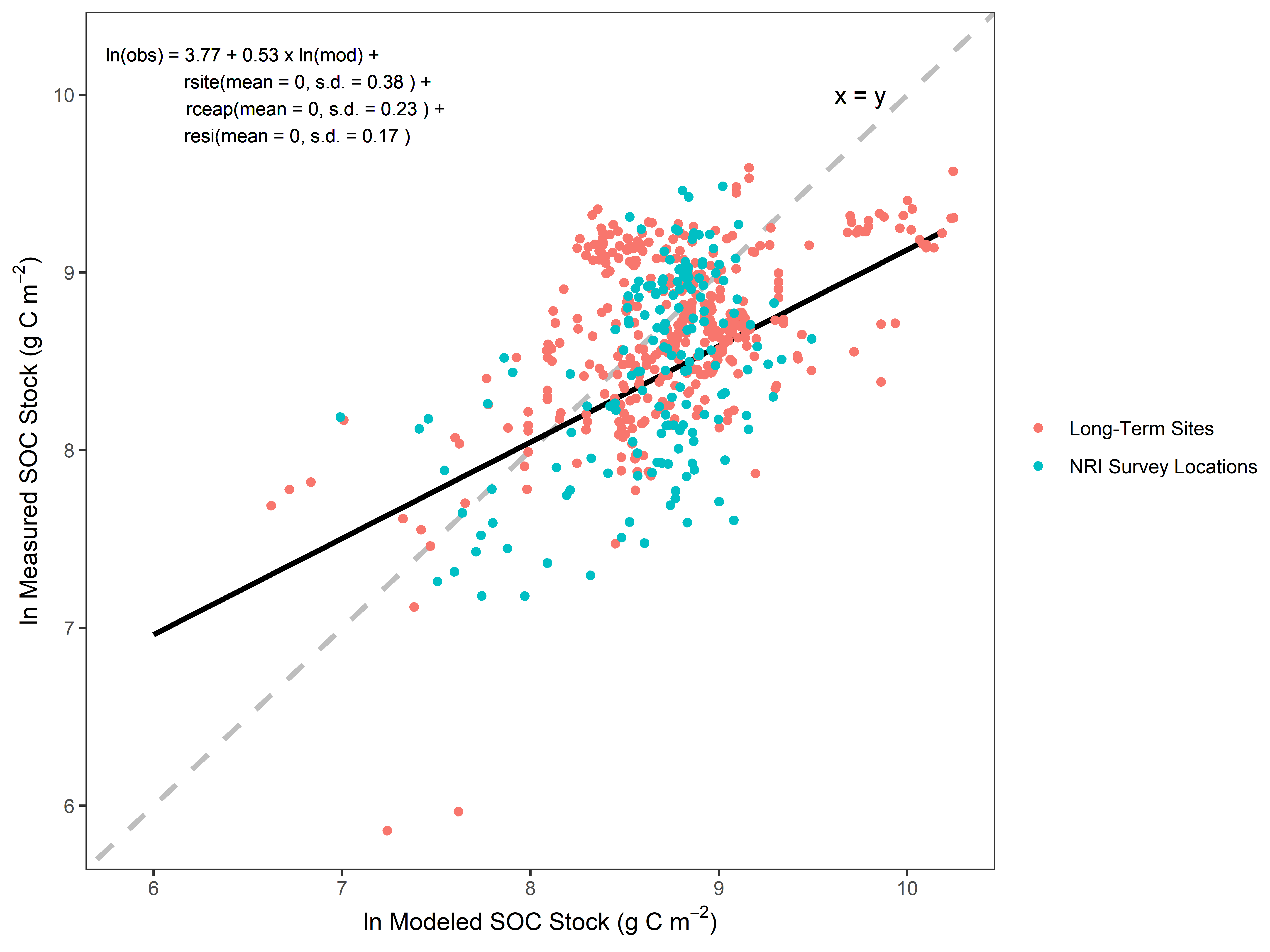


**References**

Bauer, A., and Black, A.L. 1992. Organic carbon effects on available water capacity of three soil textural groups. Soil Sci. Soc. Am. J. 56: 248-254.

Bayer C., L. Martin-Neto, J. Mielniczuk, C. N. Pillon, and L. Sangoi 2001. Changes in Soil Organic Matter Fractions under Subtropical No-Till Cropping Systems. Soil Sci. Soc. Am. J. 65:1473–1478.

Bhattacharyya T., D.K. Pala, S. Williams, B.A. Telpande, A.S. Deshmukh, P. Chandran, S.K. Ray, C. Mandal, M. Easter, K. Paustian 2010. Evaluating the Century C model using two long-term fertilizer trials representing humid and semi-arid sites from India. Agriculture, Ecosystems and Environment 139:264–272.

Black, A.L., and Tanaka, D.L. 1997. A conservation tillage-cropping systems study in the Northern Great Plains of the United States. In Soil Organic Matter in Temperate Agroecosystems. E.A. Paul, K. Paustian, E.T. Elliott, and V. Cole (eds.). CRC Press, Inc.

Bol, R., Ostle, N.J., Petzke, K.J., Chenu, C., and Balesdent, J. 2008. Amino acid 15N in long-term bare fallow soils: influence of annual N fertilizer and manure applications. European Journal of Soil Science 59: 617–629.

Brye, K.R., and Kucharik, C.J. 2003. Carbon and Nitrogen sequestration in two prairie topochronosequences on contrasting soils in southern Wisconsin. American Midland Naturalist 149:90-103.

Chivenge, P.P., Murwira, H.K., Giller, K.E., Mapfumo, P., and Six, J. 2007. Long-term impact of reduced tillage and residue management on soil carbon stabilization: Implications for conservation agriculture on contrasting soils. Soil & Tillage Research 94: 328-337. https://doi.org/10.1016/j.still.2006.08.006.

Christenson, D. R. 1997. Soil organic matter in sugar beet and dry bean cropping systems in Michigan. Chapter 10 In: Soil Organic Matter in Temperate Agroecosystems. E.A. Paul, K. Paustian, E.T. Elliott, and V. Cole (eds.). CRC Press, Inc.

Clapp, C.E., Allmaras, R.R., Layese, M.F., Linden, D.R., ans Dowdy, R.H., 2000. Soil organic carbon and 13C abundance as related to tillage, crop residue, and nitrogen fertilizer under continuous corn management in Minnesota. Soil & Tillage Research, 55: 127-142.

Clark M.S., William R. Horwath, Carol Shennan, and Kate M. Scow. 1998. Changes in Soil Chemical Properties Resulting from Organic and Low-Input Farming Practices. Agron. J. 90:662–671.

Collins, H.P., Blevins, R.L., Bundy, L.G., Christenson, D.R., Dick, W.A., Huggins, D.R., and Paul, E.A. 1999. Soil carbon dynamics in corn-based agroecosystems: Results from Carbon-13 natural abundance Soil Sci. Soc. Am. J. (1999) 63:584-591.

Collins, H.P., E.T. Elliott, K. Paustian, L.G. Bundy, W.A. Dick, D.R. Huggins, A.J.M. Smucker and E.A. Paul. 2000. Soil carbon pools and fluxes in long-term corn belt agroecosystems. Soil Biol. Biochem. 32:157-168. DOI 10.1016/S0038-0717(99)00136-4.

Denef, K., Six J., Merckx R., and Paustian K. 2004. Carbon Sequestration in Microaggregates of No-Tillage Soils with Different Clay Mineralogy. Soil Sci. Soc. Am. J. 68:1935–1944.

Dick, W.A., Edwards, W.M., and McCoy, E.L. 1997. Continuous application of no-tillage to Ohio soils: Changes in crop yields and organic matter-related soil properties. In Soil Organic Matter in Temperate Agroecosystems. E.A. Paul, K. Paustian, E.T. Elliott, and V. Cole (eds.). CRC Press, Inc.

Dolan, M.S., Clapp, C.E., Allmaras, R.R., Baker, J.M., and Molina, J.A.E., 2006. Soil organic carbon and nitrogen in a Minnesota soil as related to tillage, residue and nitrogen management. Soil & Tillage Research, 89: 221-231.

Doran, J.W., Elliott, E.T., and Paustian, K. 1998. Soil microbial activity, nitrogen cycling, and long-term changes in organic carbon pools as related to fallow tillage management. Soil & Tillage Research 49: 3-8.

Dou F., A. L. Wright, F. M. Hons 2007. Depth distribution of soil organic C and N after long-term soybean cropping in Texas. Soil & Tillage Research 94 530–536.

Elliott, E.T. I.C. Burke, C.A. Monz, S.D. Frey, K. H. Paustian, H.P. Collins, E.A. Paul, C.V. Cole, R.L. Blevins, W.W. Frye, D.J. Lyon, , A.D. Halvorson, D.R. Huggins, R.F. Turco, and M.V. Hickman. 1994. Terrestrial carbon pools in grasslands and agricultural soils: Preliminary data from the Corn Belt and Great Plains Regions. Agronomy Journal 102:990-997. DOI 10.2134/agronj2009.0483.

Follett, R.F., Paul, E.A., Leavitt, S.W., Halvorson, A.D., Lyon, D., and Peterson, G.A. 1997. Carbon isotope ratios of Great Plains soils and in wheat-fallow systems Soil Sci. Soc. Am. J. 61:1068-1077.

Franzluebbers, A.J., Hons, F.M., and Saladino, V.A. 1995. Sorghum, wheat and soybean production as affected by long-term tillage, crop sequence and N fertilization. Plant and Soil 173:55-65.

Frye, W. W., and R. L. Blevins. 1997. Soil organic matter under long-term no-tillage and conventional tillage corn production in Kentucky. . In: Soil Organic Matter in Temperate Agroecosystems. E.A. Paul. K. Paustian, E.T. Elliott, and C.V. Cole (eds.). CRC Press, Inc.

Gebhart, D.L., Johnson, H.B., Mayeux, H.S., and Polley, H.W. 1994. The CRP increases soil organic carbon. J. Soil and Water 49:488-492.

Gurung, R. B., Ogle, S. M., Breidt, F. J., Williams, S. A. & Parton, W. J. (2020) Bayesian calibration of the DayCent ecosystem model to simulate soil organic carbon dynamics and reduce model uncertainty. *Geoderma* **376**: 114529.

Hao, X., Chang, C., Travis, G.R., and Zhang, F. 2003. Soil carbon and nitrogen response to 25 annual cattle manure applications. J. Plant Nutr. Soil Sci. 166: 239-245.

Havlin, J. L., and D. E. Kissel. 1997. Management effects on soil organic carbon and nitrogen in the East-Central Great Plains of Kansas. In: Soil Organic Matter in Temperate Agroecosystems. E.A. Paul. K. Paustian, E.T. Elliott, and C.V. Cole (eds.). CRC Press, Inc.

Huggins, D. R., and D. J. Fuchs. 1997. Long-term N management effects on corn yield and soil C of an aquic haplustoll in Minnesota. In Soil Organic Matter in Temperate Agroecosystems. E.A. Paul, K. Paustian, E.T. Elliott, and V. Cole (eds.). CRC Press, Inc.

Izaurralde, R.C., McGill, W.B., Robertson, J.A., Juma, N.G., and Thurston, J.J. 2001. Carbon balance of the Breton Classical Plots over half a century. Soil Sci. Soc. Am. J. 65:431-441.

Janzen, H.H., Johnston, A.M., Carefoot, J.M., and Lindwall, C.W., 1997. Soil organic matter dynamics in long-term experiments in Southern Alberta . In Soil Organic Matter in Temperate Agroecosystems. E.A. Paul, K. Paustian, E.T. Elliott, and V. Cole (eds.). CRC Press, Inc.

Juma, N.G., Izaurralde, R.C., Robertson, J.A., and McGill, W.B. 1997. Crop yield and soil organic matter trends over 60 years in a typic cryoboralf at Breton, Alberta. In Soil Organic Matter in Temperate Agroecosystems. E.A. Paul, K. Paustian, E.T. Elliott, and V. Cole (eds.). CRC Press, Inc.

Karlen, D.L., Wollenhaupt, N.C., Erbach, D.C., Berry, E.C., Swan, J.B., Eash, N.S., and Jordahl, J.L., 1994. Crop residue effects on soil quality following 10-years of no-till corn. Soil & Tillage Research 31 149-167.

Khan, S.A., Mulvaney R.L., Ellsworth T.R., Boast C.W. 2007. The myth of nitrogen fertilization for soil carbon sequestration. J Environ Qual. 36:1821-32. doi: 10.2134/jeq2007.0099.

Kitur B. K., K.R. Olson, S.A. Ebelhar, and D.G. Bullock. 1994. Tillage effects on growth and yields of corn on Grantsburg soil. J. Soil and Water Cons. 49: 266-274.

Ludwig, B., Schulz, E., Rethemeyer, J., Merbach, I., and Flessa, H. 2007. Predictive modelling of C dynamics in the long-term fertilization experiment at Bad Lauchstadt with the Rothamsted Carbon Model. European Journal of Soil Science 58: 1155–1163.

Manies, K.L., Harden, J.W., Kramer, L., and Parton, W.J. 2001. Carbon dynamics within agricultural and native sites in the loess region of western Iowa. Global Change Biology 7: 545-555.

Manna, M.C., Swarup, A., Wanjari, R.H., and Ravankar, H.N. 2007. Long-term effects of NPK fertilizer and manure on soil fertility and a sorghum-wheat farming system. Australian Journal of Experimental Agriculture 47: 700–711.

Martens, D.A., Reedy, T.E., and Lewis, D.T. 2003. Soil organic carbon content and composition of 130-year crop, pasture and forest land-use managements. Global Change Biology 10: 65–78.

Matamala R, Jastrow JD, Miller RM, Garten CT. 2008. Temporal changes in C and N stocks of restored prairie: implications for C sequestration strategies. Ecol Appl. Sep;18(6):1470-88. doi: 10.1890/07-1609.1. PMID: 18767623.

Monreal, C. M., and H. H. Janzen. 1993. Soil organic-carbon dynamics after 80 years of cropping a Dark Brown Chernozem. . Can. J. Soil Sci. 73: 133-136. https://doi.org/10.4141/cjss93-014.

Olson, D.R., Ebelhar, S.A., and Lang, J.M. 2010. Cover crop effects on crop yields and soil organic carbon content. Soil Science 175: 89-98. DOI 10.1097/SS.0b013e3181cf795.

Paustian, K., Elliot, T. (1993) Unpublished data from soil organic carbon study funded by U.S. Environmental Protection Agency.

Peterson, G.A., and Westfall, D.G. 1997. Management of dryland agroecosystems in the central Great Plains of Colorado. In Soil Organic Matter in Temperate Agroecosystems. E.A. Paul, K. Paustian, E.T. Elliott, and V. Cole (eds.). CRC Press, Inc.

Pierce, F.J., and Fortin, M.C. 1997. Long-term tillage and periodic plowing of a no-tilled soil in Michigan: Impacts, yield, and soil organic matter. In Soil Organic Matter in Temperate Agroecosystems. E.A. Paul, K. Paustian, E.T. Elliott, and V. Cole (eds.). CRC Press, Inc.

Potter, K.N., Torbert, H.A., Johnson, H.B., and Tischler, C.R. 1999. Carbon storage after long-term grass establishment on degraded soils. Soil Science 164: 718-725.

Poulton, P. R. 1996. "The Park Grass Experiment, 1856-1995", Evaluation of soil organic matter models using existing long-term datasets. NATO ASI Series 1: Global Environmental Change, Vol 38, (Rothamsted, Harpenden, Herts UK), 377-384.

Powlson, D.S., Smith, P., Coleman, K., Smith, J.U., Glendining, M.J., Korschens, M., and Franko, U. 1998. A European network of long-term sites for studies on soil organic matter. Soil & Tillage Research 47: 263-274.

Reeder, J.D., Schuman, G.E., and Bowman, R.A. 1998. Soil C and N changes on conservation reserve program lands in the central Great Plains. Soil & Tillage Research 47:339-349.

Reedy T.E. 1999. Land use effects on the properties of a Sharpsburg soil in southeastern Nebraska. MS thesis, University of Nebraska, College of Agriculture, Lincoln, Nebraska.

Rothamsted Research (2015) Parkgrass Experiment Data Archives, 1843-2002 *Electronic Rothamsted Archive, Rothamsted Research,* https://www.era.rothamsted.ac.uk/.

Spencer, S., S.M. Ogle, F.J. Breidt, J. Goebel, and K. Paustian. 2011. Designing a national soil carbon monitoring network to support climate change policy: a case example for US agricultural lands. Greenhouse Gas Management & Measurement 1: 167-178.

Udawatta R.P., Anderson, S.H., Gantzer, C.J., and Garrett H.E. 2008. Influence of prairie restoration on CT-measured soil pore characteristics. J. Environ. Qual. 37:219–228.

USDA-NRCS (2018a) Summary Report: 2015 National Resources Inventory. Natural Resources Conservation Service, Washington, D.C., and Center for Survey Statistics and Methodology, Iowa State University, Ames, Iowa. https://www.nrcs.usda.gov/Internet/FSE_DOCUMENTS/nrcseprd1422028.pdf.

USDA-NRCS (2018b) CEAP Cropland Farmer Surveys. USDA Natural Resources Conservation Service. https://www.nrcs.usda.gov/wps/portal/nrcs/detail/national/technical/nra/ceap/na/?cid=nrcs143_014163.

Vanotti, M.B., Bundy, L.G., and Peterson, A.E., 1997. Nitrogen fertilizer and legume-cereal rotation effects on soil productivity and organic matter dynamics in Wisconsin. In Soil Organic Matter in Temperate Agroecosystems. E.A. Paul, K. Paustian, E.T. Elliott, and V. Cole (eds.). CRC Press, Inc.

Wanniarachchi, S.D., Voroney, R.P., Vyn, T.J., Beyaert, R.P., and MacKenzie, A.F. 1999. Tillage effects on the dynamics of total and corn-residue-derived soil organic matter in two southern Ontario soils. Can. J. Soil Sci. 79: 473-480.

Yang X.M., C.F. Drury, W.D. Reynolds, C.S. Tan. 2008. Impacts of long-term and recently imposed tillage practices on the vertical distribution of soil organic carbon. Soil & Tillage Research 100: 120–124.

Zhang, W.J., Wang, X.J., Xu, M.G., Huang, S.M., Liu, H., and Peng, C. 2009. Soil organic carbon dynamics under long-term fertilizations in arable land of northern China. Biogeosciences Discuss. 6: 6539–6577.
